# GAD65 Antibody Epitopes and Genetic Background in Latent Autoimmune Diabetes in Youth (LADY)

**DOI:** 10.3389/fimmu.2022.836952

**Published:** 2022-03-09

**Authors:** Yiman Peng, Xia Li, Yufei Xiang, Xiang Yan, Houde Zhou, Xiaohan Tang, Jin Cheng, Xiaohong Niu, Jing Liu, Qiuhe Ji, Linong Ji, Gan Huang, Zhiguang Zhou

**Affiliations:** ^1^ National Clinical Research Center for Metabolic Diseases, Key Laboratory of Diabetes Immunology (Central South University), Ministry of Education, and Department of Metabolism and Endocrinology, The Second Xiangya Hospital of Central South University, Changsha, China; ^2^ Department of Endocrinology, Heji Hospital Affiliated to Changzhi Medical College, Changzhi, China; ^3^ Department of Endocrinology, Gansu Provincial Hospital, Lanzhou, China; ^4^ Department of Endocrinology, Xijing Hospital, Fourth Military Medical University, Xi an, China; ^5^ Department of Endocrinology and Metabolism, Peking University People’s Hospital, Beijing, China

**Keywords:** glutamic acid decarboxylase autoantibody, GAD epitopes, HLA, type 1 diabetes (T1D), latent autoimmune diabetes in adults (LADA), latent autoimmune diabetes in youth

## Abstract

Epitope-specific GAD65Abs and *HLA-DR-DQ* gene assays help improve the value of risk stratification in autoimmune diabetes mellitus and protect islet function. Identification and early intervention are important for latent autoimmune diabetes in youth (LADY). The aims of this study were to investigate 1) the frequencies of the epitope-specific GAD65Abs and *HLA-DR-DQ* genes in LADY and 2) the association between *HLA-DR-DQ* genes and epitope-specific GAD65Abs. Higher frequencies of GAD65-CAb and multiepitope GAD65Abs were observed in young type 1 diabetes, LADY, and old type 1 diabetes subjects than those in latent autoimmune diabetes in adult (LADA) patients. The frequencies of the specific susceptible HLA haplotype *DR3*, total susceptible HLA haplotypes, and high-risk genotypes were higher in type 1 diabetes and LADY patients than those in LADA patients. In contrast, type 1 diabetes and LADY patients had lower frequencies of low/no genetic risk genotypes *(DRX/X)* than those of LADA patients. Logistic regression analysis suggested that the susceptible HLA haplotypes were risk factors for glutamic acid decarboxylase antibody (GADA) multiepitope positivity in autoimmune diabetes mellitus. LADY may be more severe than LADA, and LADY seemed to be a transitional type of type 1 diabetes and LADA. GADA epitope and *HLA-DR-DQ* gene assays are important for risk stratification in autoimmune diabetes mellitus and protection of islet function.

## Introduction

Autoimmune diabetes mellitus (ADM) is a group of highly heterogeneous autoimmune diseases characterized by autoimmune mediation and destruction of islet beta cells. In general, type 1 diabetes (T1D) is characterized by islet autoantibody positivity, juvenile onset, and the requirement for insulin therapy. In addition, subjects with phenotypic type 2 diabetes and islet antibody positivity, which has been described as “type 1.5 diabetes” (T1.5DM) or “latent autoimmune diabetes in adults (LADA)”, are non-insulin dependent for at least 6 months after onset (1). Importantly, studies have reported that 10%–75% of Caucasians and 11.7% of Chinese juvenile-onset phenotypes may have “latent autoimmune diabetes in youth (LADY)” (2, 3). Patients with LADY have a younger age of onset than those with LADA, and clinically, islet function and C-peptide levels decline more rapidly in LADY than in LADA. To date, there has been limited research on LADY, and it has not yet received attention from the international community.

Islet autoantibodies are a hallmark of ADM. The major diabetes-related autoantibodies include glutamic acid decarboxylase antibody (GADA), tyrosine phosphatase (IA-2A), zinc transporter 8 autoantibody (ZnT8A), and insulin autoantibody (IAA) (4). GADA is dominant in Western and Chinese newly diagnosed diabetes patients. Moreover, GADA has been used to screen individuals with ADM (5, 6). Epitope-specific assays of GADA may improve the clinical diagnostic specificity of diabetes patients (7).

GAD65Ab is heterogeneous with respect to its epitope specificity. Previous studies have shown the different binding patterns of GADA in T1D and LADA patients. Compared with that in T1D patients, the percentage of GAD65-NAb (N-terminal of the GAD65 protein) is significantly higher in LADA patients, while the frequency of GAD65-CAb (C-terminal of the GAD65 protein) is significantly lower in LADA patients. LADA patients with GAD65-M+Cabs (C-terminal and middle region of the GAD65 protein) have clinical features similar to those of T1D patients, and GAD65-CAb appears to confer a higher risk of the development of lower serum C-peptide levels and the requirement for insulin therapy (8–11). Schlosser et al. (12) suggested that the autoimmune response might undergo intramolecular epitope spreading progression from the N-terminal fragment to the middle fragment of GAD65 in predisposed subjects and that GAD65-CAb may be associated with the failure of islet beta cell function during disease progression.

*HLA-DRB1-DQA1* genes confer the highest risk of the occurrence of diabetes (13), and susceptible HLA genes vary among different ethnicities (14). For example, the susceptible haplotype DRB1*0901-DQA1*0302-DQB1*0303 (DR9) is more common in Chinese patients than in Caucasian patients. Furthermore, there are discrepancies in terms of susceptible HLA genotypes and haplotypes between T1D and LADA patients. For example, in Chinese patients, *DR3/DR3, DR3/DR9*, and *DR9/DR9* are T1D-associated high-risk genotypes, whereas only *DR9/DR9* is related to LADA. The haplotype DRB1*0901-DQA1*05-DQB1*0201, which confers the highest risk of T1D, is not associated with LADA. Susceptible haplotypes, including DRB1*0301-DQA1*0501-DQB1*0201 (DR3), DRB1*0405-DQA1*0303-DQB1*0401 (DR4), and DRB1*0901-DQA1*0302-DQB1*0303 (DR9), were found to be the common high-risk susceptible HLA haplotypes in T1D and LADA (15).

To date, the epitope specificity of GAD65Abs and the *HLA-DRB1-DQA1-DQB1* risk in LADY remain completely unknown, especially in large-scale research. Here, we hypothesize that LADY is a transitional type of LADA and T1D in terms of the GADA epitope and *HLA-DRB1-DQA1-DQB1* genes. We investigated the association between the *HLA-DR-DQ* genes and epitope-specific GAD65Abs. These studies could provide helpful information for understanding the pathogenesis of LADY.

## Materials and Methods

### Subjects

**Figure 1:**
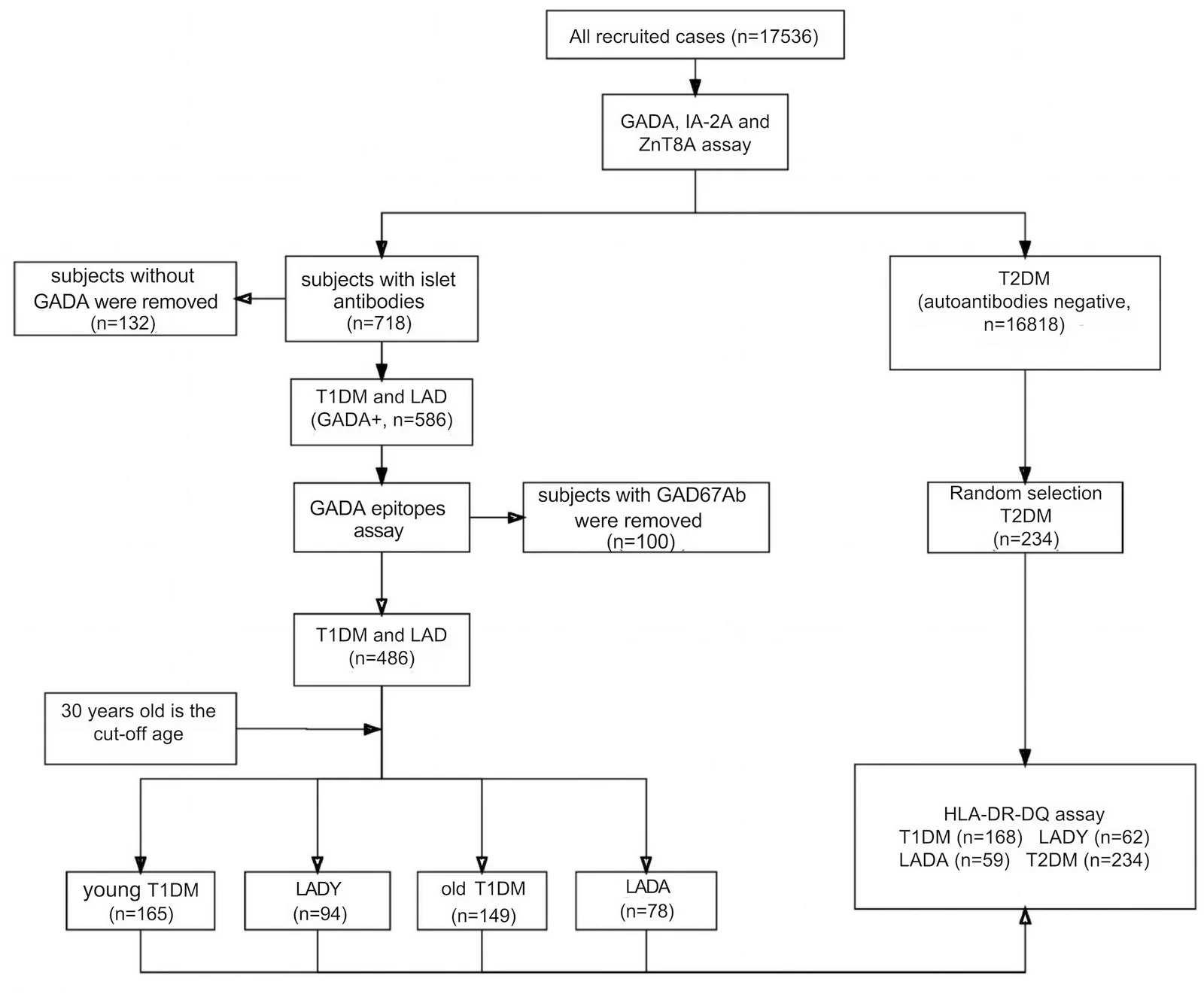
Flowchart of sampling, grouping, assaying and genotyping.

A total of 17,536 newly diagnosed diabetes mellitus patients aged 15–79 years old were recruited for this cross-sectional study from April 2015 to October 2017 (**Figure 1**). Patients from 46 different hospitals in 25 major cities were recruited consecutively to launch the “Diagnosis and Treatment Optimization of Autoimmune Diabetes in Chinese Adults” project funded by the National Key R&D Program of China (2013BAI09B12). Research staff at each participating hospital underwent standardized training on all procedures and data collection methods (16).

### Type 1 Diabetes Patients

The inclusion criteria for T1D subjects were as follows: 1) diagnosis of diabetes (World Health Organization criteria of 1999) (17) at ≥15 years of age; 2) disease duration <1 year; 3) acute onset and presence of diabetic ketosis or ketoacidosis; 4) positivity for GADA; and 5) insulin dependency at the time of diagnosis. The exclusion criteria were patients with LADY, LADA, type 2 diabetes, or a malignancy and those who were pregnant.

### Latent Autoimmune Diabetes in Youth, Latent Autoimmune Diabetes in Adults, and Type 2 Diabetes Patients

The inclusion criteria for LADY, LADA, and type 2 diabetes subjects were as follows: 1) diagnosis of diabetes (World Health Organization criteria of 1999) (17) at ≥15 years of age; 2) disease duration <1 year; 3) no ketoacidosis in the first 6 months after diagnosis of diabetes; and 4) insulin independence for at least 6 months after onset. If subjects fulfilled the above criteria, those who were autoimmune antibody (GADA, IA-2A, or ZnT8A) positive and <30 years old were diagnosed with LADY, those who were autoimmune antibody (GADA, IA-2A, or ZnT8A) positive and ≥30 years old were diagnosed with LADA, and subjects who were negative for all islet autoantibodies were diagnosed with type 2 diabetes. Patients with T1D, gestational diabetes mellitus, or malignancy and those who were pregnant were excluded. In general, LADY is distinguished from T1D by a period of at least 6 months after the onset of diabetes during which insulin is not required, LADY differs from type 2 diabetes by positivity for GADA, and 30 years old was used as the cutoff age to distinguish LADY from LADA (1).

There were 165 young T1D, 94 LADY, 149 old T1D, and 78 LADA subjects who were GADA positive assayed for epitope-specific GAD65Abs. Furthermore, 168 T1D, 62 LADY, 59 LADA, and 234 type 2 diabetes subjects were genotyped for *HLA-DRB1*, *HLA-DQA1*, and *HLA-DQB1* by direct DNA sequencing. GADA epitopes and *HLA-DRB1-DQA1-DQB1* were assayed in 289 patients, which comprised 97 young T1D, 62 LADY, 71 old T1D, and 59 LADA patients. This study was approved by the ethics committee of the Second Xiangya Hospital of Central South University, and all participants or their guardians provided written informed consent.

Physical characteristics (sex, age, height, and body weight) were recorded by professional researchers. Fasting blood was used for the measurement of fasting blood glucose (FBS), HbA1c, and fasting C-peptide (FCP). Postprandial blood samples were used to test 2-h postprandial plasma glucose (PPG) and 2-h postprandial C-peptide (PCP).

### GADA, IA-2A, and ZnT8A Assays

GADA, IA-2A, and ZnT8A were measured by radioligand binding assay in duplicate as previously described (18, 19). The cutoff values of positivity for GADA and IA-2A were 18.0 U/ml and 3.3 U/ml in World Health Organization units, and ZnT8A was positive with an antibody index of 0.011 according to the 99th percentile observed in the healthy controls. The healthy control group consisted of 405 volunteers (264 men and 141 women, mean age: 37.8 years) with normal response to the 75-g oral glucose tolerance test; they had no family history of diabetes, autoimmune diabetes, or any other chronic diseases; and they were selected for establishing the cutoff values of GADA, IA-2A, and ZnT8A assays (19, 20). The sensitivity and specificity in our laboratory were 82% and 96.7% for GADA, 76% and 100% for IA-2A, and 76% and 100% for ZnT8A, respectively, according to the Islet Autoantibody Standardization Program (IASP) 2020.

### Epitope Analysis of GAD65Ab

The epitopes of GAD65Ab were analyzed as previously described (8). The GAD65/67 chimeric constructs were responsible for the expression of fusion proteins of the N-terminal region (GAD65_1–95_/GAD67_102–593_), the middle region (GAD67_1–243_/GAD65_235–444_/GAD67_453–593_), or the C-terminal region (GAD67_1–452_/GAD65_445–585_). The levels of epitope-specific GAD65Ab were expressed as relative indices according to the 99th percentile observed in 100 local healthy controls (58 men and 42 women; mean age: 36.5 years). The cutoff values were 0.06 for the GAD67-Ab assay, 0.05 for the GAD65-NAb and GAD65-MAb assays, and 0.03 for the GAD65-CAb assay.

### *HLA-DR-DQ Genetic* Analysis

Genomic DNA was extracted from peripheral blood nucleated cells. The genotypes for *HLA-DRB1*, *HLA-DQA1*, and *HLA-DQB1* were determined by direct DNA sequencing *via* amplification of the second exon of each gene, and *DR-DQ* haplotypes were constructed by the PHASE program as described previously (15). Multiplex PCR amplifications of three HLA loci (*HLA-DRB1, HLA-DQA1*, and *HLA-DQB1*) were performed for all patients. For each donor, all HLA amplicons were pooled in a single well in approximately equimolar amounts. The samples from each individual were prepared using the Nextera XT protocol, pooled, and sequenced on a MiSeq instrument with a 2 × 250-bp paired-end cartridge (Illumina). The susceptible HLA haplotypes were DR3 (DRB1*0301-DQA1*0501-DQB1*0201), DR4 (DRB1*0405-DQA1*0303-DQB1*0401), DRB1*0405-DQA1*0301-DQB1*0302, and DR9 (DRB1*0901-DQA1*0302-DQB1*0303) (15, 21–23). The genotypes with a high genetic risk included *DR3/3, DR3/9*, and *DR9/9* (15).

### Statistical Analysis

All statistical analyses were performed using SPSS (version 26; SPSS). Two-sided *p*-values <0.05 were considered statistically significant. Data are presented as the mean ± SD or medians (25th–75th percentile). Categorical variables were compared using Fisher’s exact test or a χ^2^ test as appropriate. Continuous variables were compared using one-way ANOVA. Nonparametric tests were performed by the Mann–Whitney assay. Binary logistic regression was performed to investigate possible *HLA-DQ-DR* factors for epitope-specific GAD65Abs. A *p*-value <0.05 was considered statistically significant.

## Results

### Patterns of Epitope-Specific GAD65Abs in Young Type 1 Diabetes, Latent Autoimmune Diabetes in Youth, Old Type 1 Diabetes, and Latent Autoimmune Diabetes in Adult Patients

We used three different fragments (C-terminal, middle region, and N-terminal) to detect the GADA epitopes. Of the 586 GADA-positive patients, 100 subjects with GAD67Abs were removed to eliminate confounding factors. GAD65 epitope analysis was performed on 165 young T1D patients, 94 LADY patients, 149 old T1D patients, and 78 LADA patients. No reactivity to any of the epitopes of GAD65 was detected in 24.8%, 26.6%, 23.5%, and 41.0% of samples from young T1D patients, LADY patients, old T1D patients, and LADA patients, respectively. Further epitope analysis results are shown in **Table 1**. Compared with that in LADA subjects, the frequency of GAD65-CAb was higher in young T1D, LADY, and old T1D subjects (57.6% vs. 33.3%, *p* < 0.001; 48.9% vs. 33.3%, *p* < 0.05; and 58.4% vs. 33.3%, *p* < 0.001, respectively), and the frequency of GAD65-MAb was higher in young T1D and LADY subjects than that in LADA subjects (60.0% vs. 42.3%, *p* < 0.05; 66.0% vs. 42.3%, *p* < 0.01, respectively), whereas the frequency of GAD65-NAb was higher in LADA subjects than that in young T1D subjects (20.5% vs. 7.3%, *p* < 0.01). There was no significant difference among young T1D, LADY, and old T1D subjects in the percentage of the three different epitope-specific GAD65Abs (GAD65-CAb, GAD65-Mab, and GAD65-NAb).

**Table 1:** Prevalence of epitope-specific GAD65Ab in young T1DM, old T1DM, LADY, and LADA patients.

GADA reactivities to epitopes of GAD65	Young T1DM (n = 165)	LADY (n = 94)	Old T1DM (n = 149)	LADA (n = 78)	*p*-value
C-terminal	57.6% (95)	48.9% (46)	58.4% (87)	33.3% (26) ^†††§***^	0.001
N-region	7.3% (12)	12.8% (12)	12.8% (19)	20.5% (16) ^††^	0.031
M-terminal	60.0% (99)	66.0% (62)	54.4% (81)	42.3% (33) ^†§§^	0.012
Positive for 1 epitope	30.9% (51)	27.7% (26)	32.9% (49)	32.1% (25)	0.855
Positive for at least 2 epitopes	44.2% (73)	45.7% (43)	43.6% (65)	26.9% (21) ^†§*^	0.040

Data are expressed as % (n).

GADA, glutamic acid decarboxylase antibody.

When compared with young T1DM patients, ^†^ p < 0.05, ^††^ p < 0.01, ^†††^ p < 0.001.

When compared with LADY patients, ^§^ p < 0.05, ^§§^ p < 0.01.

When compared with old T1DM patients, ^*^ p < 0.05, ^***^ p < 0.001.

T1DM, type 1 diabetes mellitus; LADY, latent autoimmune diabetes in youth; LADA, latent autoimmune diabetes in adults.

### Frequency of GAD65Abs That Bind Different Numbers of Epitopes of GAD65 in Young Type 1 Diabetes, Latent Autoimmune Diabetes in Youth, Old Type 1 Diabetes, and Latent Autoimmune Diabetes in Adult Patients

**Figure 2:**
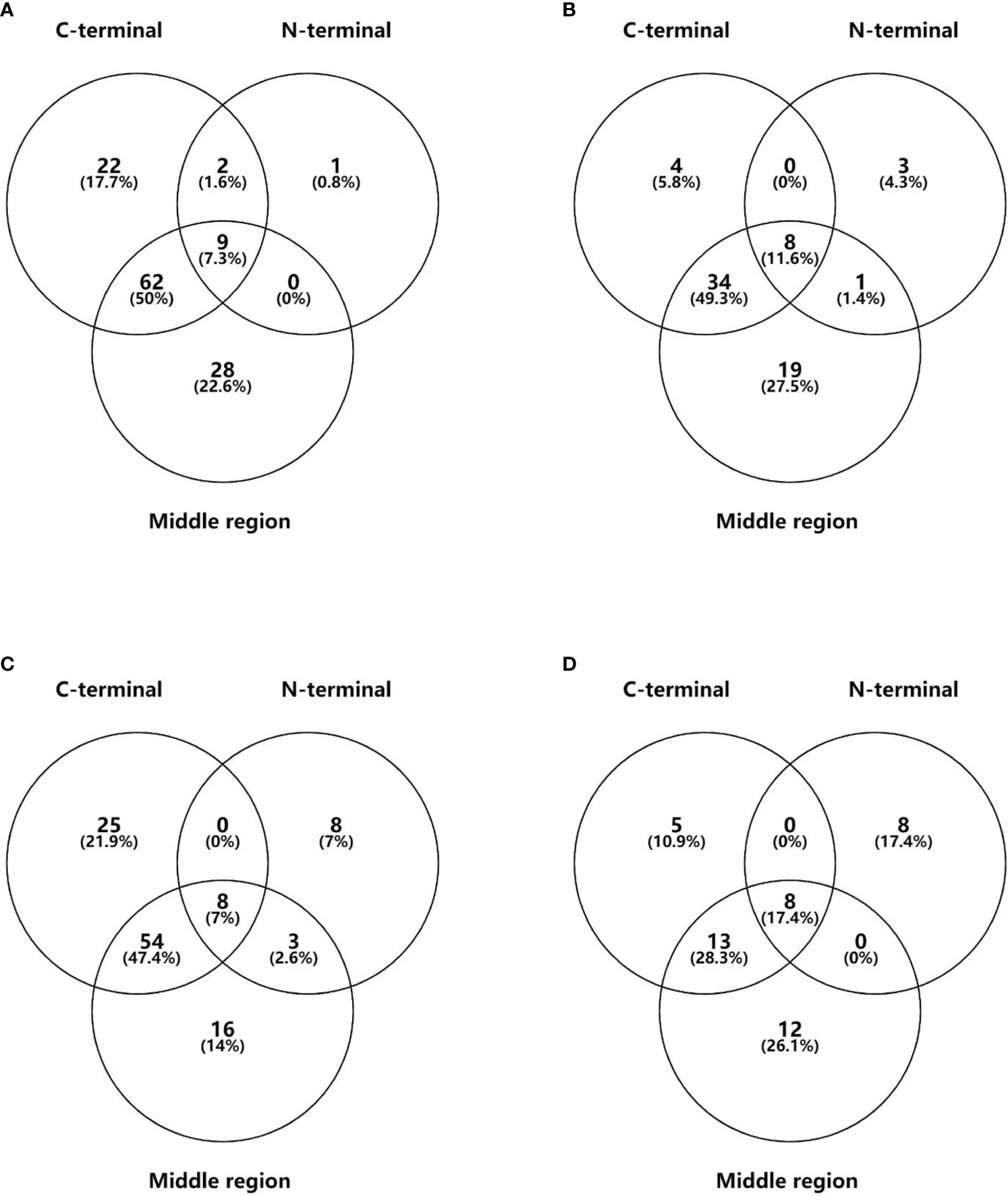
The distribution of GADA binding to different epitopes of GAD65 in young T1DM **(A)**, LADY **(B)**, old T1DM **(C)**, and LADA **(D)** patients. Data are expressed as n (%).

GADA binding to different GAD65 epitopes (N-terminal, middle region, and C-terminal) in the different groups is shown in **Table 1** and **Figure 2**. A total of 44.2% (73/165) of young T1D patients, 45.7% (43/94) of LADY patients, 43.6% (65/149) of old T1D patients, and 26.9% (21/78) of LADA patients reacted to at least two regions of GAD65. Noticeably, young T1D, LADY, and old T1D patients showed higher levels of reactivity against multiple epitopes of GAD65 than the levels of LADA patients (44.2% vs. 26.9%, *p* < 0.05; 45.7% vs. 26.9%, *p* < 0.05; and 43.6% vs. 26.9%, *p* < 0.05, respectively). There was no significant difference among young T1D, LADY, and old T1D patients in the frequency of GAD65Ab that binds different numbers of epitopes of GAD65.

### Clinical Characteristics of Young Type 1 Diabetes, Latent Autoimmune Diabetes in Youth, Old Type 1 Diabetes, and Latent Autoimmune Diabetes in Adult Patients

As shown in **Table 2**, the levels of FCP were higher in LADY and LADA subjects than those in young T1D and old T1D subjects; furthermore, compared with levels in LADA patients, LADY patients had lower levels of FCP. The level of PCP manifested a similar trend; compared with levels in young T1D and old T1D patients, LADY and LADA patients had a higher level of PCP, but there was no significant difference between LADY and LADA patients in the level of PCP. GADA titers were significantly different only between old T1D and LADA patients [old T1D vs. LADA: 376.8 (118.4–902.6) vs. 180.4 (50.3–450.3) U/ml, *p* < 0.01].

**Table 2:** Comparison of clinical features of patients with young T1DM, LADY, old T1DM, and LADA.

Variable	Young T1DM (n = 165)	LADY (n = 94)	Old T1DM (n = 149)	LADA (n = 78)	*p*-value
Age (years)	23 ± 4	25 ± 4^†^	47 ± 11^†††§§§^	52 ± 12^†††§§§**^	<0.001
Female/male	72/93	33/61	53/96	36/42	0.231
BMI (kg/m^2^)	19.8 ± 3.5	21.3 ± 3.6^††^	22.0 ± 3.7^†††^	22.5 ± 3.3^†††§^	<0.001
SBP (mmHg)	116 ± 13	116 ± 14	120 ± 15^††§^	123 ± 13^†††§§^	<0.001
DBP (mmHg)	74 ± 9	74 ± 9	77 ± 11^†^	79 ± 10^††§§^	0.004
FBS (mmol/L)	9.8 ± 4.7	9.2 ± 4.3	9.7 ± 4.1	9.6 ± 4.2	0.746
HbA_1C_ (mmol/mol)	105.6 ± 37.8	100.5 ± 38.2	98.8 ± 33.1	88.9 ± 31.9^††^	0.008
HbA_1C_ (%)	11.8 ± 3.5	11.3 ± 3.5	11.2 ± 3.0	10.3 ± 2.9^††^	0.008
FCP (pmol/L)	120 (60–202)	230 (143–380) ^†††^	122 (53–239) ^§§§^	423 (223–655) ^†††§***^	<0.001
PCP (pmol/L)	190 (96–395)	490 (289–815) ^†††^	208 (114–463) ^§§§^	1,070 (442–1747) ^†††***^	<0.001
TG (mmol/L)	0.94 (0.72–1.49)	1.10 (0.79–1.74)	1.03 (0.70–1.65)	1.37 (0.93–2.07) ^††^	0.013
TC (mmol/L)	4.30 ± 1.22	4.48 ± 1.39	4.46 ± 1.61	4.64 ± 1.22	0.379
LDL-C (mmol/L)	2.58 ± 0.91	2.79 ± 1.08	2.61 ± 1.04	2.74 ± 1.34	0.386
HDL-C (mmol/L)	1.21 (0.99–1.48)	1.11 (0.96–1.40)	1.17 (0.97–1.50)	1.16 (0.96–1.42)	0.341
GAD65Ab (U/ml)	269.2 (97.2–757.2)	267.7 (84.5–600.5)	376.8 (118.4–902.6)	180.4 (50.3–450.3) ^**^	0.007

Data are presented as the mean ± SD, median (IQR), or ratio.

SBP, systolic blood pressure; DBP, diastolic blood pressure; FBS, fasting blood glucose; FCP, fasting C peptide; PCP, 2-h postprandial C peptide; TG, triglycerides; TC, total cholesterol; HDL-C, HDL cholesterol; LDL-C, LDL cholesterol.

When compared with young T1DM patients, ^†^ p < 0.05, ^††^ p < 0.01, ^†††^ p < 0.001.

When compared with LADY patients, ^§^ p < 0.05, ^§§^ p < 0.01, ^§§§^ p < 0.001.

When compared with old T1DM patients, ^**^ p < 0.01, ^***^ p < 0.001.

T1DM, type 1 diabetes mellitus; LADY, latent autoimmune diabetes in youth; LADA, latent autoimmune diabetes in adults.

### *HLA-DR-DQ* Haplotype and Genotype Frequency Analysis

As shown in **Table 3**, the frequency of the susceptible HLA haplotype DR3 was higher in T1D and LADY patients than that in LADA patients (T1D vs. LADA: 16.7% vs. 6.8%, *p* < 0.01; LADY vs. LADA: 26.6% vs. 6.8%, *p* < 0.001). Similarly, the frequencies of total susceptible HLA haplotypes were significantly higher in T1D and LADY patients than that in LADA patients (T1D vs. LADA: 58.6% vs. 37.3%, *p* < 0.001; LADY vs. LADA: 64.5% vs. 37.3%, *p* < 0.001). Moreover, the frequencies of high-risk genotypes were higher in T1D and LADY patients than that in LADA patients (T1D vs. LADA: 25.0% vs. 10.2%, *p* < 0.05; LADY vs. LADA: 35.5% vs. 10.2%, *p* < 0.01). In contrast, T1D and LADY patients had lower frequencies of low/no genetic risk genotypes (*DRX/X*) than the frequencies of LADA patients (T1D vs. LADA: 14.9% vs. 40.7%, *p* < 0.001; LADY vs. LADA: 14.5% vs. 40.7%, *p* < 0.01). Unexpectedly, LADY patients had a higher frequency of the susceptible haplotype *DR3* than that of T1D patients (26.6% vs. 16.7%, *p* < 0.05). There were no significant differences between LADY and T1D patients in the frequencies of total susceptible HLA haplotypes, high-risk genotypes, and low/no genetic risk genotypes (*DRX/X)*. There was no difference in HLA genes between LADA and type 2 diabetes patients.

**Table 3:** The frequency of susceptible HLA haplotypes and genotypes among T1DM, LADY, LADA, and T2DM subjects.

Variable	T1DM	LADY	LADA	T2DM	*p*-value
n	168	62	59	234	N/A
Onset age	32 ± 13	25 ± 4^†††^	54 ± 11^†††§§§^	36 ± 15^§§§***^	<0.001
Female/male	67/101	23/39	28/31	82/152	0.339
*DR3*	16.7 (56)	26.6 (33) ^†^	6.8 (8) ^††§§§^	5.8 (27) ^†††§§§^	<0.001
*DR4*	12.5 (42)	11.3 (14)	7.6 (9)	4.5 (21) ^†††§§^	<0.001
DRB1*04:05-DQA1*03:01-DQB1*03:02	0.3 (1)	0.8 (1)	1.7 (2)	0.2 (1)	0.181
*DR9*	29.2 (98)	25.8 (32)	21.2 (25)	18.8 (88) ^††^	0.006
Total susceptible haplotypes	58.6 (197)	64.5 (80)	37.3 (44) ^†††§§§^	29.3 (137) ^†††§§§^	<0.001
*DR3/3, DR-3/9, DR-9/9* (high genetic risk)	25.0 (42)	35.5 (22)	10.2 (6) ^†§§^	5.1 (12) ^†††§§§^	<0.001
DRX/X (no/low genetic risk)	14.9 (25)	14.5 (9)	40.7 (24) ^†††§§^	51.3 (120) ^†††§§§^	<0.001

Data are presented as the mean ± SD or % (n).

^†^Compared with T1DM p < 0.05, ^††^ Compared with T1DM p < 0.01, ^†††^ Compared with T1DM p < 0.001.

^§§^Compared with LADY p < 0.01, ^§§§^ Compared with LADY p < 0.001.

^*^Compared with LADA p < 0.05, ^***^ Compared with LADA p < 0.001.

DR3, DRB1*03:01-DQA1*05:01-DQB1*02:01; DR4, DRB1*04:05-DQA1*03:03-DQB1*04:01; DR9, DRB1*09:01-DQA1*03:02-DQB1*03:03; Total susceptible haplotypes, DR3+DR4+DRB1*04:05-DQA1*03:01-DQB1*03:02+DR9; X, other than DR3, DR4, DRB1*04:05-DQA1*03:01-DQB1*03:02, and DR9; LADY, latent autoimmune diabetes in youth; LADA, latent autoimmune diabetes in adults; T1DM, type 1 diabetes mellitus; T2DM, type 2 diabetes mellitus.

### Correlation Between the *HLA-DR-DQ* Genes and Epitope-Specific GAD65Ab

Because the appearance, positivity, and affinity of GADA are related to the *HLA-DR-DQ* genes, as reported in previous studies (24–27), we tried to investigate the association between epitope-specific GAD65Abs (GAD65-CAb, GAD65-Mab, and GAD65-NAb) and the *HLA-DR-DQ* gene. As shown in **Table 4**, the susceptible HLA haplotype was a risk factor for GAD65Ab multiepitope positivity (r = 1.900; *p* < 0.05). Among the susceptible HLA haplotypes shared by T1D and LADA patients, *DR3* conferred the highest genetic susceptibility (15). We further investigated the association between different common susceptible HLA haplotypes and GAD65Ab multiepitope positivity. Logistic regression analysis suggested that patients with *DR3* had a higher risk of GAD65Ab multiepitope positivity (r = 1.763; *p* < 0.05). In addition, GAD65-CAb was considered to be related to worse beta-cell function and greater insulin therapy demand (7, 8, 20). We analyzed the association between *HLA-DR-DQ* genes and GAD65-CAb but found no significant association between them (**Table 5**).

**Table 4:** Association of the frequency of GADA binding to multiple epitopes of GAD65 with the frequencies of *HLA-DR-DQ* haplotypes and genotypes.

Variable	OR (95% CI)	*p*-value
*DR3*	1.76 (1.04–2.99)	0.035*
*DR4*	1.22 (0.69–2.16)	0.499
*DR9*	0.81 (0.50–1.31)	0.384
Susceptible haplotypes	1.90 (1.02–3.54)	0.043*
*DR3/DR3, DR3/DR9*, and *DR9/DR9*	1.23 (0.71–2.12)	0.457

DR3, DRB1*03:01-DQA1*05:01-DQB1*02:01; DR4, DRB1*04:05-DQA1*03:03-DQB1*04:01; DR9, DRB1*09:01-DQA1*03:02-DQB1*03:03; susceptible haplotypes, DR3+ DR4+ DR9.

*Represents p value <0.05, which is statistically significant.

**Table 5:** Association of the frequency of GAD65-CAb with the frequencies of *HLA-DR-DQ* haplotypes and genotypes.

Variable	OR (95% CI)	*p*-value
*DR3*	1.49 (0.88–2.52)	0.139
*DR4*	1.16 (0.66–2.04)	0.612
*DR9*	0.90 (0.56–1.44)	0.649
Susceptible haplotypes	1.56 (0.88–2.79)	0.130
*DR3/DR3, DR3/DR9*, and *DR9/DR9*	1.18 (0.69–2.02)	0.555

DR3, DRB1*03:01-DQA1*05:01-DQB1*02:01; DR4, DRB1*04:05-DQA1*03:03-DQB1*04:01; DR9, DRB1*09:01-DQA1*03:02-DQB1*03:03; susceptible haplotypes, DR3+ DR4+DR9.

## Discussion

GADA has been used to screen individuals with autoimmune diabetes. Over the past few decades, reports about the epitope specificity of GAD65Abs in T1D and LADA have increased (8, 11). Autoimmune diabetes is considered to be a continuous spectrum (28). For the first time, we disclosed the epitope-specific GAD65Ab frequencies from young T1D, LADY, old T1D, and LADA patients, but especially in LADY patients. We also found an association between the *HLA-DR-DQ* gene and epitope-specific GAD65Abs. LADY seems to be a transitional type of T1D and LADA. This finding suggests the important value of the identification and early intervention in protecting islet function for LADY. Clinically, it is necessary to protect the islet function of LADY patients as early as possible in the window period. These results provide helpful information for understanding the pathogenesis of LADY.

Compared with T1D patients, a lower frequency of GAD65-CAb and a higher frequency of GAD65-Nab were observed in LADA patients, and the discrepancy in GAD65 epitope patterns between T1D and LADA suggests different immune activities toward islet beta cells. As the results showed, LADY may be more severe than LADA, and the percentage of GAD65-CAb was significantly higher in young T1D, LADY, and old T1D subjects than that in LADA subjects, which reveals the important value of epitope-specific assays in the identification process and islet function protection. Furthermore, compared with those of LADA patients, the levels of FCP were observed to be lower in LADY patients, and GAD65-CAb and multiepitope GAD65Ab positivity were considered to be related to worse islet function (7, 8). In terms of epitope reactivity, young T1D, LADY, and old T1D patients showed higher reactivities to multiple epitopes of GAD65 than did LADA patients, which indicates a stronger or broader immune response. These findings suggested that there may be more severe immune damage progression in young T1D, LADY, and old T1D patients than in LADA patients. Epitope-specific assays of GAD65Abs may be useful in predicting the need for insulin therapy in autoimmune diabetes.

The *DRB1, DQA1*, and *DQB1* loci were highly associated with diabetes susceptibility (3, 15, 29, 30). *HLA-DR-DQ* genes in T1D, LADY, LADA, and type 2 diabetes patients were compared for the first time in this study, especially between LADY and LADA patients. The higher frequencies of total susceptible HLA haplotypes and high-risk genotypes in T1D and LADY manifested an increased HLA genetic susceptibility load compared with that of LADA, which may imply a shared pathogenesis between T1D and LADY, suggesting that the similarity in the frequencies of the epitope-specific GAD65Abs between T1D and LADY subjects may be derived from similar HLA genetic backgrounds. *DR3*, total susceptible haplotypes, and high-risk HLA genotypes were more frequent in LADY than those in LADA, and the discrepancy in HLA genes between LADY and LADA indicated an increased HLA genetic susceptibility load in LADY patients, LADY was likely to be more severe than LADA, and early clinical intervention and different therapeutic strategies to preserve islet function were required for LADY patients.

T1D and LADY subjects had a higher susceptible HLA risk and higher reactivity to GAD65Ab than LADA subjects. An association between HLA genes and the affinity of GAD65Ab has been reported (16, 24, 25). This study investigated the association between *HLA-DR-DQ* genes and the epitope specificity of GAD65Abs, which may be useful in revealing the interaction between HLA and the diabetes autoimmune response. HLA gene risk and GAD65 epitope specificity may accurately stratify the risk of ADM patients. Susceptible HLA haplotypes were a risk factor for GADA multiepitope positivity, especially *DR3*. HLA genes affected GADA multiepitope positivity, which is an immunoreactive process with epitope expansion within the GADA molecule. This finding has an implication similar to that of a previous study by Pöllänen et al. (31). They reported that HLA genes affected positivity for multiple islet autoantibodies, and patients with high-risk HLA genotypes had a greater tendency to be positive for multiple antibodies than that of patients with moderate-risk HLA genotypes (31). Transitioning from single antibody positivity to multiple antibody positivity reflects an expansion of the immune response between different islet autoantibody molecules. The above two findings revealed that HLA contributed to both the intermolecular and intramolecular expansion of islet autoimmunity.

LADY seems to be more closely related to T1D than LADA. First, the percentage of GAD65-CAb was higher in young T1D, LADY, and old T1D subjects than that in LADA subjects. Second, young T1D, LADY, and old T1D patients showed a higher frequency of multiepitope GAD65Abs than that in LADA patients. Third, the frequencies of total susceptible HLA haplotypes and high-risk genotypes were higher in T1D and LADY subjects than those in LADA subjects, while the frequency of no/low genetic risk genotypes (*DRX/X*) was higher in LADA subjects than those in T1D and LADY subjects. Last, the levels of FCP and PCP were similarly lower in T1D and LADY subjects than those in LADA subjects, which suggested similar islet function in T1D and LADY patients. These findings are of great importance in clinical prevention, judgment, and the use of insulin in LADY patients.

In summary, T1D and LADY patients had more similar HLA-DR-DQ genetic backgrounds and epitope-specific GAD65Abs than LADA patients. The discrepancies in terms of the frequencies of susceptible HLA genes between LADY and LADA may contribute to the different manifestations of epitope-specific GAD65Abs; LADY was likely to be more severe than LADA. This information could be useful for classifying LADY patients and could provide novel insight into understanding the pathology underlying LADY. In addition, the existence of susceptible HLA haplotypes was a risk factor for the frequency of GAD65Ab multiepitope positivity, especially *DR3*, revealing the intricate pathogenesis of autoimmune diabetes in terms of the HLA genetic background and GADA immune response. Whereas the number of individuals with LADY and LADA in this study was relatively small, there was no long-term observation of the level of C-peptide and the frequencies of epitope-specific GAD65Abs, which may be dynamic during the progression of disease. It will be necessary to explore more specific and detailed results in large-scale and follow-up cohort studies to provide novel insight into the pathogenesis of autoimmune diabetes.

## Data Availability Statement

The original contributions presented in the study are included in the article/**Supplementary Material**. Further inquiries can be directed to the corresponding author.

## Ethics Statement

The studies involving human participants were reviewed and approved by the ethics committee of the Second Xiangya Hospital of Central South University. Written informed consent to participate in this study was provided by the participants’ legal guardian/next of kin.

## Author Contributions

YP conducted the experiments, analyzed the data, and wrote the article. GH and ZZ conceived and designed the study. YP and GH analyzed and interpreted the results. XL, YX, XY, HZ, XT, JC, XN, JL, QJ, and LJ researched the data. All the authors critically revised the article and approved the final version. GH is the guarantor of this work and, as such, had full access to all the data in the study and takes responsibility for the integrity of the data and the accuracy of the data analysis.

## Funding

This study was supported by the National Key R&D Program of China (2018YFC1315600, 2013BAI09B12 and 2016YFC1305001), the National Natural Science Foundation of China (81820108007 and 81873634).

## Conflict of Interest

The authors declare that the research was conducted in the absence of any commercial or financial relationships that could be construed as a potential conflict of interest.

## Publisher’s Note

All claims expressed in this article are solely those of the authors and do not necessarily represent those of their affiliated organizations, or those of the publisher, the editors and the reviewers. Any product that may be evaluated in this article, or claim that may be made by its manufacturer, is not guaranteed or endorsed by the publisher.

## Abbreviations

ADM, Autoimmune diabetes mellitus; DBP, Diastolic blood pressure; FBS, Fasting blood glucose; FCP, Fasting C-peptide; GADA, GAD autoantibodies; GAD-C, C-terminal of the GAD65 protein; GAD-M, Middle region of the GAD65 protein; GAD-N, N-terminal of the GAD65 protein; GAD65, 65-kDa isoform of GAD; GAD67, 67-kDa isoform of GAD; HDL-C, High-density lipoprotein cholesterol; IAA, Insulin autoantibodies; IA-2A, Protein tyrosine phosphatase autoantibodies; LADA, Latent autoimmune diabetes in adults; LADY, Latent autoimmune diabetes in youth; LDL-C, Low-density lipoprotein cholesterol; PCP, 2-h postprandial C-peptide; SBP, Systolic blood pressure; TC, Total cholesterol; TG, Triglycerides; ZnT8A, Zinc transporter 8 autoantibodies.

## Appendix

The members of the National Clinical Research Center for Metabolic Diseases (investigators and hospitals): Xueyao Han, Ling Chen, and Xiaoling Chen, Peking University People’s Hospital; Lixin Guo, Xiaofan Jia, and Shan Ding, Beijing Hospital; Xinhua Xiao, Cuijuan Qi, and Xiaojing Wang, Peking Union Medical College Hospital; Zhongyan Shan, Yaxin Lai, and Zhuo Zhang, The First Hospital of China Medical University; Yu Liu, Yan Cheng, and Hanqing Cai, The Second Hospital of Jilin University; Yadong Sun, Yan Ma, and Haiying Wang, People’s Hospital of Jilin Province; Yiming Li, Chaoyun Zhang, and Shuo Zhang, Hua Shan Hospital, Fudan University; Tao Yang, Hao Dai, and Mei Zhang, The First Affiliated Hospital of Nanjing Medical University; Liyong Yang, Peiwen Wu, and Xiaofang Yan, The First Affiliated Hospital of Fujian Medical University; Yangang Wang, Fang Wang, and Hong Chen, The Affiliated Hospital of Qingdao University; Qifu Li and Rong Li, The First Affiliated Hospital of Chongqing Medical University; Li Wang and Xiangyang Liu, Xijing Hospital, Fourth Military Medical University; Suhong Wei, Gansu Provincial Hospital; Yun Zhu and Rui Ma, The First Affiliated Hospital of Xinjiang Medical University; Gebo Wen, Xinhua Xiao, and Jianping Qin, The First Affiliated Hospital of the University of South China; Jian Kuang and Yan Lin, Guangdong General Hospital; Shaoda Lin and Kun Lin, the First Affiliated Hospital of Shantou University Medical College; Li Li, Heji Hospital Affiliated with Changzhi Medical College; Gan Huang and Shuoming Luo, The Second Xiangya Hospital of Central South University; Huibiao Quan and Leweihua Lin, Hainan General Hospital; Hongyu Kuang and Weihua Wu, The First Affiliated Hospital of Harbin Medical University; Yuling He, The First Affiliated Hospital of Guangxi Medical University; Xiaoyan Chen and Yuyu Tan, The First Affiliated Hospital of Guangzhou Medical University; Ling He, Guangzhou First People’s Hospital; Chao Zheng, The Second Affiliated Hospital of Wenzhou Medical University; Jianying Liu and Zhifang Yang, The First Affiliated Hospital of Nanchang University; Xiaoyang Lai, The Second Affiliated Hospital of Nanchang University; Ling Hu, Yan Zhu and Ying Hu, The Third Affiliated Hospital of Nanchang University; Xuqing Li, Henan Provincial People’s Hospital; Hong Li and Yushan Xu, The First Affiliated Hospital of Kunming Medical University; Heng Su and Yang Ou, The First People’s Hospital of Yunnan Province; Jianping Wang, The Second Hospital University of South China; Changqing Luo and Xiaoyue Wang, The First People’s Hospital of Yueyang; Zhiming Deng and Shenglian Gan, The First People’s Hospital of Changde City; Zhaohui Mo, Ping Jin and Honghui He, The Third Xiangya Hospital of Central South University; Qiuxia Huang, Dongguan People’s Hospital; Fang Wang, Heping Hospital Affiliated with Changzhi Medical College; Yi Zhang and Zhenzhen Hong, First Hospital of Quanzhou Affiliated with Fujian Medical University; Yuezhong Ren and Pengfei Shan, The Second Affiliated Hospital of Zhejiang University School of Medicine; Caifeng Yan and Hui Zhang, Northern Jiangsu People’s Hospital; Zhiwen Liu, Shanghai Xuhui District Central Hospital; Meibiao Zhang, The First People’s Hospital of Huaihua; Ming Liu and Heting Wang, Tianjin Medical University General Hospital; Hongwei Jiang and Liujun Fu, The First Affiliated Hospital of Henan University of Science and Technology; Hui Fang, Tangshan Gongren Hospital; and Hui Sun, The Affiliated Hospital of Inner Mongolia Medical University.
