# Correction: GAD65 antibody epitopes and genetic background in latent autoimmune diabetes in youth (LADY)

**DOI:** 10.3389/fimmu.2026.1966969

**Published:** 2026-09-02

**Authors:** Yiman Peng, Xia Li, Yufei Xiang, Xiang Yan, Houde Zhou, Xiaohan Tang, Jin Cheng, Xiaohong Niu, Jing Liu, Qiuhe Ji, Linong Ji, Gan Huang, Zhiguang Zhou

**Affiliations:** 1 National Clinical Research Center for Metabolic Diseases, Key Laboratory of Diabetes Immunology (Central South University), Ministry of Education, and Department of Metabolism and Endocrinology, The Second Xiangya Hospital of Central South University, Changsha, China; 2 Department of Endocrinology, Heji Hospital Affiliated to Changzhi Medical College, Changzhi, China; 3 Department of Endocrinology, Gansu Provincial Hospital, Lanzhou, China; 4 Department of Endocrinology, Xijing Hospital, Fourth Military Medical University, Xi’an, China; 5 Department of Endocrinology and Metabolism, Peking University People’s Hospital, Beijing, China

**Keywords:** glutamic acid decarboxylase autoantibody, GAD epitopes, HLA, type 1 diabetes (T1D), latent autoimmune diabetes in adults (LADA), latent autoimmune diabetes in youth

In the published article, there was an error in **Table 1** as published. “GADA reactivities to epitopes of GAD65” in **Table 1** was improperly named. “N-region” should be corrected to “N-terminal”, and “M-terminal” to “Middle region”. The corrected **Table 1** and its caption appear below.

**Table 1:** Prevalence of epitope-specific GAD65Ab in young T1DM, old T1DM, LADY and LADA patients.

GADA reactivities to epitopes of GAD65	Young T1DM (n=165)	LADY (n=94)	Old T1DM (n=149)	LADA (n=78)	*p* value
C-terminal	57.6% (95)	48.9% (46)	58.4% (87)	33.3% (26) ^†††§***^	0.001
N-terminal	7.3% (12)	12.8% (12)	12.8% (19)	20.5% (16) ^††^	0.031
Middle region	60.0% (99)	66.0% (62)	54.4% (81)	42.3% (33) ^†§§^	0.012
Positive for 1 epitope	30.9% (51)	27.7% (26)	32.9% (49)	32.1% (25)	0.855
Positive for at least 2 epitopes	44.2% (73)	45.7% (43)	43.6% (65)	26.9% (21) ^†§*^	0.040

Data are expressed as % (n).

GADA, glutamic acid decarboxylase antibody.

When compared with young T1DM patients, ^†^*p* < 0.05, ^††^*p* < 0.01, ^†††^*p* < 0.001.

When compared with LADY patients, ^§^*p* < 0.05, ^§§^*p* < 0.01, ^§§§^*p* < 0.001.

When compared with old T1DM patients, ^*^p < 0.05, ^**^p < 0.01, ^***^p < 0.001.

In the published article, there was an error in **Figure 1** as published. “Young T1D” should be changed to “young T1DM”, “wereremoved” should be changed to “were removed” and “old T1dM” should changed to “old T1DM”. The corrected **Figure 1** and its caption appear below.

**Figure 1:**
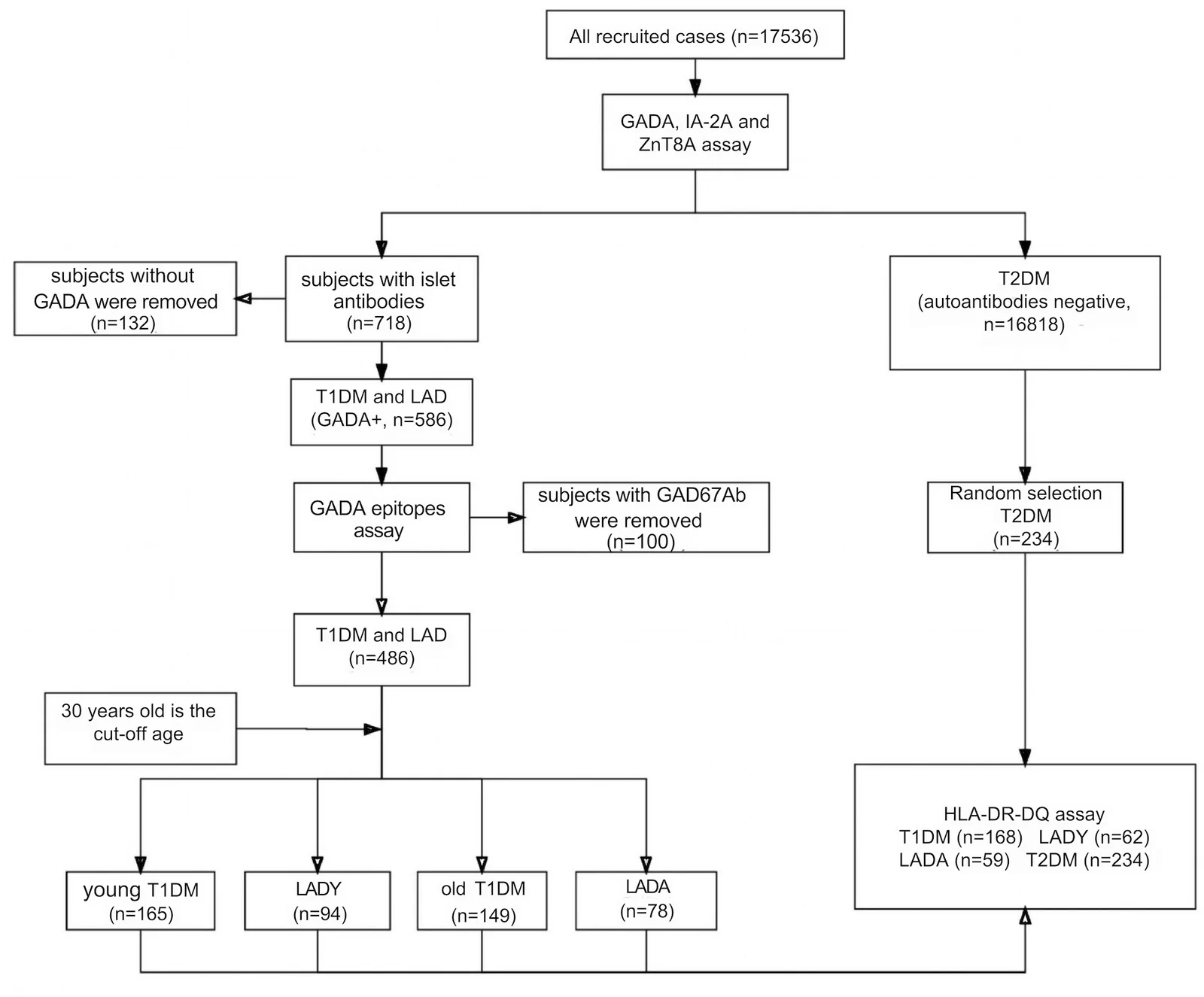
Flowchart of sampling, grouping, assaying and genotyping.

In the published article, there was an error. A correction has been made to **Materials and Methods**, *HLA -DR-DQ Genetic Analysis*. This sentence previously stated:

“The susceptible HLA haplotypes were DR3 (DRB1*0301-DQA1*0501-DQB1*0201), DR4 (DRB1*0405-DQA1*0303-DQB1*0401), DRB1*0405-DQA1*0301-DQB1*0302, and DR9 (DRB1*0901-DQA1*0303-DQB1*0302) (15, 21–23).”

The corrected sentence appears below:

“The susceptible HLA haplotypes were DR3 (DRB1*0301-DQA1*0501-DQB1*0201), DR4 (DRB1*0405-DQA1*0303-DQB1*0401), DRB1*0405-DQA1*0301-DQB1*0302, and DR9 (DRB1*0901-DQA1*0302-DQB1*0303) (15, 21-23).”

The original article has been updated.

