# Supplementary material for: GAD65 Antibody Epitopes and Genetic Background in Latent Autoimmune Diabetes in Youth (LADY)
Source: Front Immunol. 2022 Mar 9;13:836952. doi: 10.3389/fimmu.2022.836952 (PMC8982141; doi:10.3389/fimmu.2022.836952)
Supplement: Supplementary file 1 [file Table_1.docx]

**Supplemental Table 1** Association of the frequency of GAD65-CAb with the frequencies of *HLA-DR-DQ* haplotypes and genotypes in young T1DM, LADY, old T1DM and LADA.

|  | young T1DM |  |  | LADY |  |  | old T1DM |  |  | LADA |  |
| --- | --- | --- | --- | --- | --- | --- | --- | --- | --- | --- | --- |
|  | OR (95% CI) | *P^a^* |  | OR (95% CI) | *P^a^* |  | OR (95% CI) | *P^a^* |  | OR (95% CI) | *P^a^* |
| *DR3* | 1.36 (0.57-3.23) | 0.484 |  | 1.67 (0.60-4.64) | 0.326 |  | 0.82 (0.24-2.76) | 0.753 |  | 2.12 (0.39-11.60) | 0.387 |
| *DR4* | 0.56 (0.22-1.43) | 0.225 |  | 0.60 (0.17-2.09) | 0.423 |  | 2.69 (0.77-9.38) | 0.121 |  | 2.92 (0.6912.40) | 0.147 |
| *DR9* | 1.58 (0.67-3.73) | 0.297 |  | 0.55 (0.19-1.57) | 0.265 |  | 0.66 (0.26-1.70) | 0.391 |  | 0.69 (0.22-2.17) | 0.522 |
| Susceptibility haplotypes | 1.26 (0.39-4.08) | 0.699 |  | 1.50 (0.38-5.94) | 0.564 |  | 0.97 (0.28-3.42) | 0.964 |  | 1.44 (0.47-4.38) | 0.526 |
| *DR3/DR3*, *DR3/DR9* and *DR9/DR9* | 1.39 (0.50-3.88) | 0.526 |  | 1.11 (0.39-3.13) | 0.851 |  | 0.37 (0.13-1.03) | 0.058 |  | 12.67 (1.36-117.64) | 0.026 |

**Supplemental Table 2** Association of the frequency of GADA binding to GAD65 multiple epitopes with the frequencies of *HLA-DR-DQ* haplotypes and genotypes in young T1DM, LADY, old T1DM and LADA.

|  | young T1DM |  |  | LADY |  |  | old T1DM |  |  | LADA |  |
| --- | --- | --- | --- | --- | --- | --- | --- | --- | --- | --- | --- |
|  | OR (95% CI) | *P^a^* |  | OR (95% CI) | *P^a^* |  | OR (95% CI) | *P^a^* |  | OR (95% CI) | *P^a^* |
| *DR3* | 1.75 (0.75-4.09) | 0.194 |  | 2.09 (0.75-5.87) | 0.161 |  | 0.59 (0.16-2.22) | 0.417 |  | 3.08 (0.55-17.15) | 0.200 |
| *DR4* | 0.44 (0.16-1.19) | 0.105 |  | 0.71 (0.20-2.47) | 0.586 |  | 3.16 (1.00-10.02) | 0.051 |  | 4.43 (1.01-19.38) | 0.048 |
| *DR9* | 1.47 (0.64-3.37) | 0.368 |  | 0.38 (0.13-1.11) | 0.077 |  | 0.85 (0.33-2.19) | 0.734 |  | 0.51 (0.14-1.85) | 0.305 |
| Susceptibility haplotypes | 2.05 (0.58-7.16) | 0.263 |  | 1.29 (0.32-5.10) | 0.721 |  | 1.47 (0.40-5.43) | 0.563 |  | 1.74 (0.52-5.88) | 0.371 |
